# Role of microbial interactions in the impaired cultivability of thermophilic lactic acid bacteria in natural whey starter for Parmigiano Reggiano PDO cheese production

**DOI:** 10.3389/fmicb.2026.1755652

**Published:** 2026-03-04

**Authors:** Marianna Cristofolini, Maria Ronsivalle, Maria Pramazzoni, Giulia Zaccarini, Valentina Pizzamiglio, Lisa Solieri

**Affiliations:** 1 Lactic Acid Bacteria and Yeasts Biotechnology (LYB) Lab, Department of Life Sciences, University of Modena and Reggio Emilia, Reggio Emilia, Italy; 2 Dipartimento di Scienze Agrarie, Alimentari e Agro-ambientali, University of Pisa, Pisa, Italy; 3 Consorzio del Formaggio Parmigiano Reggiano, Reggio Emilia, Italy

**Keywords:** cultivability, Lactobacillus delbrueckii, Lactobacillus helveticus, microbial interactions, mutualism, natural whey starter, Streptococcus thermophilus

## Abstract

Natural whey starter (NWS) cultures play a pivotal role in the production of Parmigiano Reggiano (PR) Protected Designation of Origin (PDO) cheese; however, their microbial ecology and functional dynamics remain only partially understood. In particular, *Lactobacillus delbrueckii* subsp. *lactis*, a dominant species in type-D NWS communities, exhibits impaired cultivability that limits its isolation and characterization. Consequently, most studies have focused on strain variability within *Lactobacillus helveticus*, which is predominant in type-H NWS communities. In this study, we evaluated the effects of 14 different medium supplementations on the recovery and maintenance of *L. delbrueckii* subsp. *lactis* isolates from two PR NWS samples representatives of type-D and type-H communities. Although most supplementations increased lactobacilli plate counts compared with the control MRS medium, they failed to sustain cell viability during the purification for culture collection establishment. Moreover, these media altered species ratios in favor of *L. helveticus*, even when *L. delbrueckii* dominated the community according to metagenomic profiling (type-D NWS). Supplementation of MRS medium with cysteine and formic acid enabled the recovery of viable *L. delbrueckii* subsp. *lactis* isolates, accounting for 35% of the strains obtained from type-D NWS. Cross-feeding experiments further revealed that co-culturing *L. delbrueckii* with the formate-producing *Streptococcus thermophilus* significantly enhanced milk acidification compared with monocultures, indicating a beneficial metabolic interaction. In contrast, no such improvement was observed in the presence of *L. helveticus*, likely due to negative interactions with *L. delbrueckii* subsp. *lactis.* Accordingly, the impaired cultivability of *L. delbrueckii* subsp. *lactis* could thus be partially alleviated either in co-culture with *S. thermophilus* or under axenic conditions mimicking natural metabolite exchange between these species.

## 1 Introduction

Parmigiano Reggiano (PR) cheese is one of the most renowned Italian raw-milk, hard-cooked, Protected Designation of Origin (PDO) cheeses. During its production, natural whey starter (NWS) cultures play a crucial role in shaping both the sensory and rheological characteristics of the final product (Neviani et al., 2024). These starters consist of complex consortia of thermophilic lactic acid bacteria (LAB), primarily *Lactobacillus helveticus*, *Lactobacillus delbrueckii* subsp. *lactis*, *Limosilactobacillus fermentum*, and *Streptococcus thermophilus* (Cocconcelli et al., 1997; Coppola et al., 2000; Gatti et al., 2003, 2004; Sola et al., 2022).

NWS cultures were produced through continuous back-slopping, whereby sweet whey from the previous cheesemaking batch is incubated for approximately 20 h under controlled conditions, with a gradually decreasing temperature following curd cooking. Upon inoculation into milk, NWS microorganisms rapidly dominate the microbial community and drive intense lactic fermentation (De Dea Lindner et al., 2008, Gatti et al., 2008). The lactic acid produced, in synergy with the action of calf rennet, promotes casein coagulation, lowers the curd pH, and enhances whey expulsion, processes that are essential for proper curd formation and texture development (Cogan et al., 2007; Gobbetti and Di Cagno, 2017). During the early stages of cheesemaking, primary proteolysis, mainly driven by rennet activity, predominates, while proteolytic enzymes released upon microbial cell lysis contribute to secondary proteolysis and to the formation of several flavor-active compounds, particularly those derived from branched-chain amino acids (Gatti et al., 2008).

Despite the central role of NWS cultures in PR cheese production, their microbial ecology remains complex and only partially understood. Several culture-independent studies have characterized the microbial composition of NWS (Bottari et al., 2010; De Filippis et al., 2014; Alessandria et al., 2016; Morandi et al., 2019; Bertani et al., 2020; Morandi et al., 2022). More recently, two major PR NWS types have been defined based on the dominant species: type-H, dominated by *L. helveticus*, and type-D, dominated by *L. delbrueckii* (Sola et al., 2022). However, no studies have systematically investigated how the microbial composition of type-H and type-D NWS influences curd acidification and, consequently, the sensorial attributes of the final cheese. Moreover, the abiotic or biotic parameters influencing the establishment of one NWS type over the other remain largely unknown.

Culture-dependent studies have been fragmented and primarily focused on *L. helveticus* fraction (Gatti et al., 1999; Giraffa and Neviani, 1999; Giraffa et al., 2000; Lombardi et al., 2002; Gatti et al., 2003, 2004), while *L. delbrueckii* and *S. thermophilus* populations have been largely overlooked. To date, the only exception was a study which compared the acidifying and proteolytic activities of *L. delbrueckii* subsp. *lactis* strains from different dairy sources, including PR NWS (Giraffa et al., 2004).

Culture-based approaches offer several advantages over sequencing-based methods, especially for biotechnological applications, as they enable the isolation and preservation of bacterial strains for further genetic and phenotypic characterization (Almeida et al., 2019). These methods are essential for investigating microbial functionality at the strain level, particularly in the context of dairy fermentation, and for establishing collections of pure cultures that are critical for the bottom-up assembly of synthetic microbial communities (SMC) (Rodríguez Amor and Dal Bello, 2019; Nikoloudaki et al., 2024). As simplify representations of complex microbial communities, SMCs represent a valuable tool to study resistance, resilience, and functional interactions in microbial communities like NWS (Nikoloudaki et al., 2024). However, in the case of PR NWS, the isolation of pure cultures from starter communities has proven particularly challenging. Sola et al. (2022) reported a marked decline in the cultivability of NWS isolates, especially those belonging to *L. delbrueckii*, which failed to grow on conventional LAB isolation media such as MRS. Similarly, Morandi et al. (2019) described the inability to isolate *L. delbrueckii* from Grana Padano NWS. This limited cultivability hampers the study of intraspecies variability and microbial interactions under controlled conditions, thereby hindering the reconstruction of simplified SMC that mimic NWS consortia. Consequently, our understanding of how variations in NWS species or strains abundance affect cheese quality remains limited.

To investigate the factors underlying the poor cultivability of NWS lactobacilli and of *L. delbrueckii* in particular, the present study examined the role of culture media composition and microbial interactions. Specifically, 14 different media formulations were evaluated for their ability to (i) maximize recovery of viable plate counts, (ii) maintain cultivability of axenic cultures during culture collection establishment, and (iii) preserve species relative abundances present in mixed microbial communities. In parallel, microbial interactions were investigated through cross-feeding experiments in milk using different combinations of *S. thermophilus*, *L. delbrueckii* subsp. *lactis*, and *L. helveticus*.

## 2 Materials and methods

### 2.1 Reagents, cultivation media, and LAB strains

Unless otherwise stated, media and anaerobic systems were purchased from Oxoid (Basingstoke, Hampshire, United Kingdom), while the chemicals were purchased from Sigma Aldrich (St. Louis, MO, United States). Primers and Sanger sequencing were provided by BMR Genomics (Padova, Italy), while the molecular biology reagents by Thermo Fisher Scientific (Waltham, MA, United States).

Type strains *L. helveticus* DSM 20075*^T^*, *L. fermentum* DSM 20052*^T^*, *L. delbrueckii* subsp. *bulgaricus* DSM 20081*^T^*, *L. delbrueckii* subsp. *lactis* DSM 20073*^T^*, and *S. thermophilus* DSM 20176*^T^* served as reference in species attribution and were cultured according to the DSM guidelines. For cross-feeding experiments, the strains *S. thermophilus* RBC06 and C001.27, *L. helveticus* C001.15 and LBB04, and *L. delbrueckii* subsp. *lactis* T1104 and C5I3 were originally isolated from NWS using M17 and MRS media, as specified in Supplementary Figure 1. All strains were subsequently deposited in the culture collection of the Lactic Acid Bacteria and Yeast Biotechnology (LYB) Laboratory of the Department of Life Sciences (University of Modena and Reggio Emilia). Strains were routinely propagated under the same isolation conditions, as detailed in Supplementary Table 1.

### 2.2 Sampling, vitality, and physicochemical characterization of fresh NWS

NWS samples were collected in winter 2023 from two PR dairies located in the province of Reggio Emilia (Italy), both belonging to the Parmigiano Reggiano PDO Cheese Consortium. The samples, previously classified as type-H and type-D (Sola et al., 2022), were aseptically collected immediately after overnight whey incubation and stored under refrigerated conditions until analysis. All analyses were performed on the same day as sampling.

Total microbial counts (TMC) were determined using a Bürker chamber by counting cells in at least 10 randomly selected quadrants under a Nikon ECLIPSE 80i microscope at 400 × magnification. Cell viability in NWS samples was assessed using the LIVE/DEAD BacLight Bacterial Viability Kit (Thermo Fisher Scientific). Detailed protocols for TMC determination and viability assessment are reported in Cristofolini et al. (2025).

Titratable acidity (expressed in Soxhlet-Henkel degrees, °SH/50 mL), pH, and fermentative activity (defined as acidification rate and expressed as Δ°SH/50 mL) were determined following the procedures described by Cristofolini et al. (2025).

### 2.3 Total DNA extraction and metagenome sequencing

Bacterial cells were harvested from 2 mL of each NWS sample by centrifugation at 9,000 g for 10 min. After cell washing with TE buffer (10 mM Tris-HCl, 1 mM EDTA, pH 8.0), total DNA was extracted using the DNeasy PowerSoil 96 Pro Kit (Qiagen, Hilden, Germany) on a QIAcube HT instrument (Qiagen, Hilden, Germany). The integrity and concentration of the extracted DNA were assessed by 1.5% agarose gel electrophoresis. Libraries were prepared and subjected to Illumina NovaSeq X platform by BMR Genomics (Padua, Italy). Specifically, libraries were prepared using the Illumina DNA Prep kit with IDT for Illumina UD Indexes, with adapter trimming performed using CTGTCTCTTATACACATCT, i7 adapters CA AGCAGAAGACGGCATACGAGAT[i7]GTCTCGTGGGCTCGG, and i5 adapters AATGATACGGCGACCACCGAGATCTACAC[i5] TCGTCGGCAGCGTC. Library quality was evaluated by measuring DNA concentration with a Qubit fluorometer (Thermo Fisher Scientific, Waltham, MA, United States) and fragment size distribution using a Bioanalyzer (Agilent Technologies, Santa Clara, CA, United States). Libraries were pooled by mass and sequenced on an Illumina NovaSeq X platform in paired-end 150 bp mode (2 × 150 bp). Raw sequencing reads in FASTQ format were preprocessed with fastp to remove adapters and low-quality bases, using a qualified Phred score of 20, an unqualified base limit of 30%, an average quality threshold of 25, and low-complexity filtering with a complexity threshold of 30 (Chen et al., 2018). Filtered reads were aligned with Bowtie (v.2.2.3) (Langmead et al., 2009) using default parameters against the SILVA v138 ribosomal RNA database (Quast et al., 2013) and the *Homo sapiens* GRCh38 genome to remove potential host contamination. Taxonomic profiling of microbial communities was then performed using MetaPhlAn v4 (June 2023 database) (Blanco-Míguez et al., 2023). Metataxonomic profile datasets generated for this study have been deposited in the NCBI GenBank database under the BioProject accession number PRJNA1367500.

### 2.4 Culturomics analyses

Media used for culturomics analyses were listed in Table 1. MRS and M17 media were prepared according to the manufacturer’s instructions, with bacteriological agar added at a final concentration of 1.5% (w/v) when required. M17 and MRS media were supplemented with sterile skimmed whey (SSW; Reire, Reggio Emilia, Italy) at a final concentration of 3.5 g/L. A 5% (w/v) SSW stock solution was prepared as described by Fornasari et al. (2006). Yeast extract (YE) and tryptone (TRYPTO) were added to MRS at final concentrations of 1, 2, and 4% (w/v) before autoclaving. Lactalbumin and casein hydrolysates were prepared as 10% (w/v) stock solutions, sterilized, and added to MRS medium at 1, 2 and 4% (w/v), after autoclaving. L-cysteine (C) was added to MRS medium prior to autoclaving at a final concentration of 0.5 g/L, while formic acid (F) was aseptically added after autoclaving to MRS medium at a final concentration of 5 mM. Folic acid (Fa) was prepared as a 50 μg/mL stock solution in ddH_2_O, filter-sterilized (0.2 μm), and added to MRS medium after autoclaving at a final concentration of 226 μM.

**TABLE 1:** Media and incubation conditions used in the present study.

Abbreviation	Composition (supplements added to basal medium)	Incubation conditions (O_2_, temperature; time)
CTRL	MRS medium (Peptone 1%, Beef extract 0.8%, Yeast extract 0.4%, Glucose 2%, Tween 80 0.001%, K_2_HPO_4_ 0.2%, Sodium acetate trihydrate 0.5%, Ammonium citrate 0.2%, MgSO_4_⋅7H_2_O 0.02%, MnSO_4_⋅4H_2_O 0.005%)	Anaerobiosis; 42°C; 48–72 h
M17-SWW	M17 medium (Tryptone 0.5%, Soy peptone 0.5%, Beef extract 0.5%, Yeast extract 0.25%, Ascorbic acid 0.05%, MgSO_4_ 0.025%, Disodium β-glycerophosphate 1.9%, Lactose 0.5%) + skimmed sweet whey (SSW) 7%	Aerobiosis; 42°C; 72 h
YPDA	Yeast extract 1%, Peptone 2%, Glucose 2%, Agar 2%	Aerobiosis; 27°C; 48 h
SWW	MRS + skimmed sweet whey (SSW) 7% (w/v)	Anaerobiosis; 42°C; 48–72 h
YE	MRS + yeast extract 4%	Anaerobiosis; 42°C; 48–72 h
1% TRYPTO	MRS + tryptone 1%	Anaerobiosis; 42°C; 48–72 h
2% TRYPTO	MRS + tryptone 2%	Anaerobiosis; 42°C; 48–72 h
4% TRYPTO	MRS + tryptone 4%	Anaerobiosis; 42°C; 48–72 h
1% CAS	MRS + casein hydrolysate 1%	Anaerobiosis; 42°C; 48–72 h
2% CAS	MRS + casein hydrolysate 2%	Anaerobiosis; 42°C; 48–72 h
4% CAS	MRS + casein hydrolysate 4%	Anaerobiosis; 42°C; 48–72 h
1% LACTO	MRS + lactalbumin hydrolysate 1%	Anaerobiosis; 42°C; 48–72 h
2% LACTO	MRS + lactalbumin hydrolysate 2% (w/v)	Anaerobiosis; 42°C; 48–72 h
4% LACTO	MRS + lactalbumin hydrolysate 4% (w/v)	Anaerobiosis; 42°C; 48–72 h
C	MRS + cysteine 4.3 mM	Anaerobiosis; 42°C; 48–72 h
CF	MRS + cysteine 4.3 mM + Formic acid 5 mM	Anaerobiosis; 42°C; 48–72 h
CFFa	MRS + cysteine 4.3 mM + formic acid 5 mM + folatic acid 226 μM	Anaerobiosis; 42°C; 48–72 h

Concentrations are expressed as% (w/v), where “w/v” indicates grams of solute per 100 mL of solution, unless otherwise specified. Concentrations of cysteine, formic acid, and folic acid are expressed in mM or μM. CTRL, control; YE, MRS supplemented with yeast extract; SSW, MRS supplemented with skimmed sweet whey; TRYPTO; MRS supplemented with tryptone; CAS, MRS supplemented with 2% casein hydrolysate; 4% CAS, MRS supplemented with casein hydrolysate; LACTO, MRS supplemented with 4% lactalbumin hydrolysate; C, MRS supplemented with cysteine; CF, MRS supplemented with cysteine and formic acid; CFFa, MRS supplemented with cysteine, formic acid, and folic acid.

All media used for bacterial enumeration were supplemented with cycloheximide (50 μg/mL) to inhibit yeasts growth.

YPDA medium was prepared with 1% (w/v) yeast extract, 2% (w/v) peptone, 2% (w/v) glucose, and 1.5% (w/v) agar, to monitor generalist yeasts as contaminants in NWS. Chloramphenicol (50 μg/mL) was added to inhibit bacterial growth.

NWS samples were tenfold serially diluted in sterile saline solution (9 g/L NaCl) and plated by surface spreading using a sterile L-shaped spreader. Plates were incubated according to incubation conditions reported in Table 1. Specifically, lactobacilli were enumerated by incubating the plates at 42°C for 48–72 h under anaerobic conditions; streptococci were enumerated by incubating M17-SSW plates at 42°C for 72 h under aerobic conditions, as described by Fornasari et al. (2006); yeasts were enumerated by incubating YPDA plates at 42°C for 48 h under aerobic conditions. Viable cell counts were recorded as colony-forming units (CFU/mL) from plates containing 20–200 colonies and expressed as Log_10_ CFU/mL, calculated as the mean of at least three replicates.

Individual colonies were randomly selected from plates containing 20–200 colonies and sub-cultured through three successive rounds of purification on the same isolation medium. The number of viable and cultivable isolates was monitored at each round of streaking and compared to the initial number of colonies selected from the primary plates to assess survival. Cultivability was expressed as the percentage of surviving colonies after three rounds of purification, according to Equation 1:


$$\text{Cultivability}\left(\%\right)=\frac{\left(N\,v-N\,d\right)}{N\,i}*100$$ (1)

where N_*d*_ is the number of colonies that died during the three rounds of purification; N_*v*_ is the number of surviving colonies after three rounds of purification; N_*i*_ is the number of colonies initially isolated from the starting plate.

Axenic strains were examined for catalase activity, Gram-staining, and morphology, and subsequently stored at −80 °C in liquid medium supplemented with 25% (v/v) glycerol.

### 2.5 LAB species identification

Genomic DNA was extracted by mechanical lysis from LAB isolates harvested at the early stationary phase, followed by organic solvent extraction, and quantified spectrophotometrically, as previously reported (Tagliazucchi et al., 2020). LAB isolates were identified by pentaplex PCR according to Cremonesi et al. (2011), with the exceptions that primer concentrations were reduced to 1 μmol/L and PCR conditions followed those described by Martini et al. (2024). When required, 16S amplified ribosomal DNA restriction analysis (16S-ARDRA) using the diagnostic endonucleases *Mse*I and *Eco*RI and 16S rRNA gene sequencing were performed as previously described (Giraffa et al., 1998; Sola et al., 2022).

### 2.6 Growth experiments

*Lactobacillus delbrueckii* subsp. *lactis* strains C5I3 and T1104 were grown on the corresponding isolation medium (Supplementary Table 1) until the late exponential phase and then inoculated into 5 mL of MRS, M17-SSW, C, CF, or CFFa media at a final cell suspension of app. 10^5^ CFU/mL. Cultures were incubated at 42°C for 72 h under anaerobic conditions, after which growth was monitored by measuring optical density at 600 nm (OD_600_) using a microplate reader (Multiskan SkyHigh, Thermo Fisher Scientific, United States). Growth was expressed as percentage relative to the OD_600_ values of the control condition (MRS medium), as reported by Fontana et al. (2019). All growth experiments were carried out in triplicate.

### 2.7 Cross-feeding experiments

**FIGURE 1:**
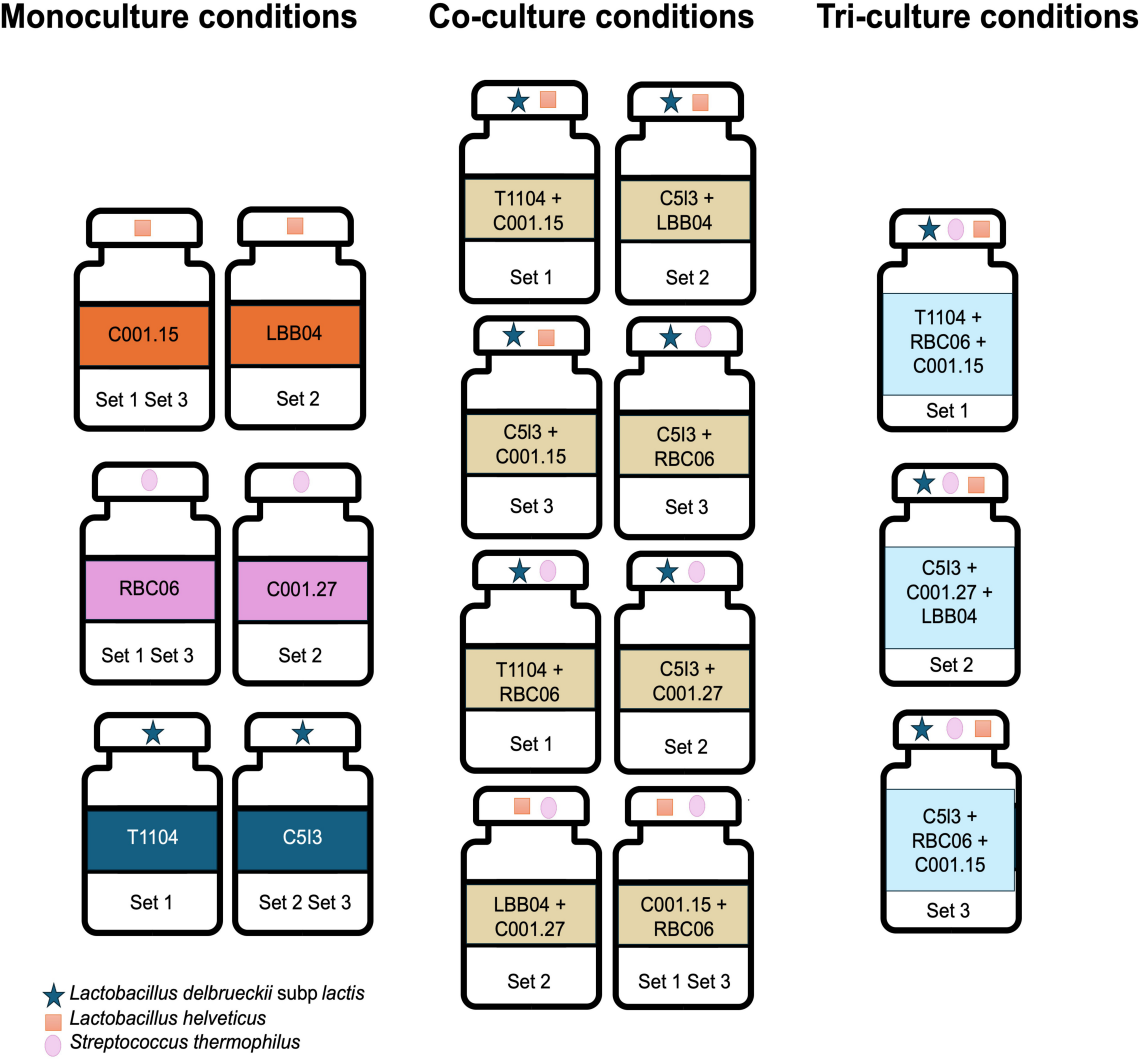
Experimental design of cross-feeding trails in milk.

Cross-feeding experiments were conducted in milk using three combinations of six NWS-derived strains, including two strains of *S. thermophilus* (St), two strains of *L. helveticus* (Lh), and two strains of *L. delbrueckii* subsp. *lactis* (Ld). Milk fermentations were performed using single monocultures (St, Lh, and Ld), 1:1 co-cultures (St + Lh, St + Ld, and Lh + Ld), and 1:1:1 tri-cultures (St + Lh + Ld), resulting in a total of 17 inoculation conditions. The experimental design of the cross-feeding tests is illustrated in Figure 1. Specifically, individual strains were initially propagated in tubes containing 5 mL of the corresponding isolation medium at 42°C for 48 h according to the culture conditions described in Supplementary Table 1. Pre-cultures were scaled up to 35 mL under the same conditions and incubated until reaching the stationary phase. Cell densities were spectrophotometrically quantified at 600 nm (OD_600_), using species-specific calibration curves to correlate OD values with viable cell counts (CFU/mL) (Rutella et al., 2016). Prior to inoculation, appropriate volumes of each culture were centrifuged (6,000 rpm, 10 min, 4°C), and cell pellets were resuspended in 1 mL of sterile physiological solution. Cell suspensions were then inoculated in 40 mL of partially skimmed UHT milk (Parmalat, Reggio Emilia, Italy), previously aseptically aliquoted in 100 mL screw-cap Erlenmeyer flaks, to achieve a final concentration of app. 2 × 10^7^ CFU/mL. For co-culture and tri-culture fermentations, cell suspensions of the respective species were combined in 1:1 or 1:1:1 ratio immediately prior to inoculation to reach the same final concentration (2 × 10^7^ CFU/mL). The total inoculum size was kept constant across all conditions to avoid variability in acidification rate due to differences in inoculum density. Uninoculated milk was included as a negative control in each experiment. All milk fermentation assays were performed in triplicate. Inoculated milk samples were incubated at 42°C for 160 h in a temperature-controlled water bath.

### 2.8 Milk acidification and lactic acid determination

Milk acidification was monitored three times per day (at 9:00, 13:00, and 17:00) by aseptically collecting 800 μL of sample and measuring pH using a penetration pH meter (XS Instruments, Carpi, MO, Italy). Fermentations were considered complete when the pH remained constant for at least three consecutive measurements. Changes in pH (ΔpH), calculated as the difference between the initial pH (pH_0_) and the pH measured at each time point, were plotted against time to generate the acidification curves. Acidification curves were modeled using the gcFitSpline function available in *grofit* R package (Kahm et al., 2010) to calculate maximum acidification rate (μ_*max*_. pH units h^–1^) and maximum acidification efficiency (expressed as ΔpH_*max*_).

At the end of fermentation, fermented milk samples were centrifuged (9,000 rpm, 10 min, 4°C), and the supernatants were 0.45 μm-filtered before enzymatic determination of D- and L-lactic acid concentrations, according to the manufacturer’s instructions (Cat. No.: K-DLATE; Megazyme, Wicklow, Ireland). Enzymatic measurements were performed using a microplate reader (Multiskan SkyHigh, Thermo Fisher Scientific, United States).

### 2.9 Statistical analysis

Statistical analyses were performed using GraphPad Prism software (v.10, GraphPad Software, La Jolla, CA, United States). Data were analyzed by two-way ANOVA, followed by Tukey’s multiple comparison test as *post hoc* analysis, unless otherwise specified. Differences were considered statistically significant at *p* < 0.05 and, where applicable, are indicated by different letters.

## 3 Results

### 3.1 NWS characterization and metataxonomic profiling

The physicochemical and microbiological properties of the two NWS samples analyzed in this study are reported in Table 2. TMC values exceeded 9 Log_10_ cells/mL in both samples, with viability values of 90.4% for type-D NWS and 89.5% for type-H NWS. Lactobacilli counts on conventional MRS medium were 8.05 Log_10_ CFU/mL for type-D NWS and 8.01 Log_10_ CFU/mL for type-H NWS. In both samples, streptococci (evaluated on M17-SSW medium) and contaminant yeasts (evaluated on YPDA medium) were present, in agreements with previous reports (Martini et al., 2021; Sola et al., 2022). Overall, the data indicate that the NWS samples are high dense cultures of viable and metabolically active LAB cells.

**TABLE 2:** Physicochemical and microbiological properties of the two NWS samples considered in this study.

Properties	NWS type-H	NWS type-D
Ph	3.54 ± 0.01	3.40 ± 0.01
Fermentative activity (Δ°SH/50 mL) (44°C)	8.52 ± 0.04	7.39 ± 0.03
Titratable acidity (Δ°SH)	28.6 ± 0.10	33.00 ± 0.13
Lactobacilli counts (Log_10_ CFU/mL)	8.01 ± 0.06	8.05 ± 0.01
Streptococci counts (Log_10_ CFU/mL)	7.41 ± 0.01	6.40 ± 0.006
Generalist yeast counts (Log_10_ CFU/mL)	2.69 ± 0.02	1.81 ± 0.01
TMC (Log_10_ Cells/mL)	9.13 ± 0.05	9.27 ± 0.07
Viability (%)	89.5 ± 3.3	90.40 ± 7.3

Data are presented as means of three replicates ± standard deviation. TMC, total microbial counts.

**FIGURE 2:**
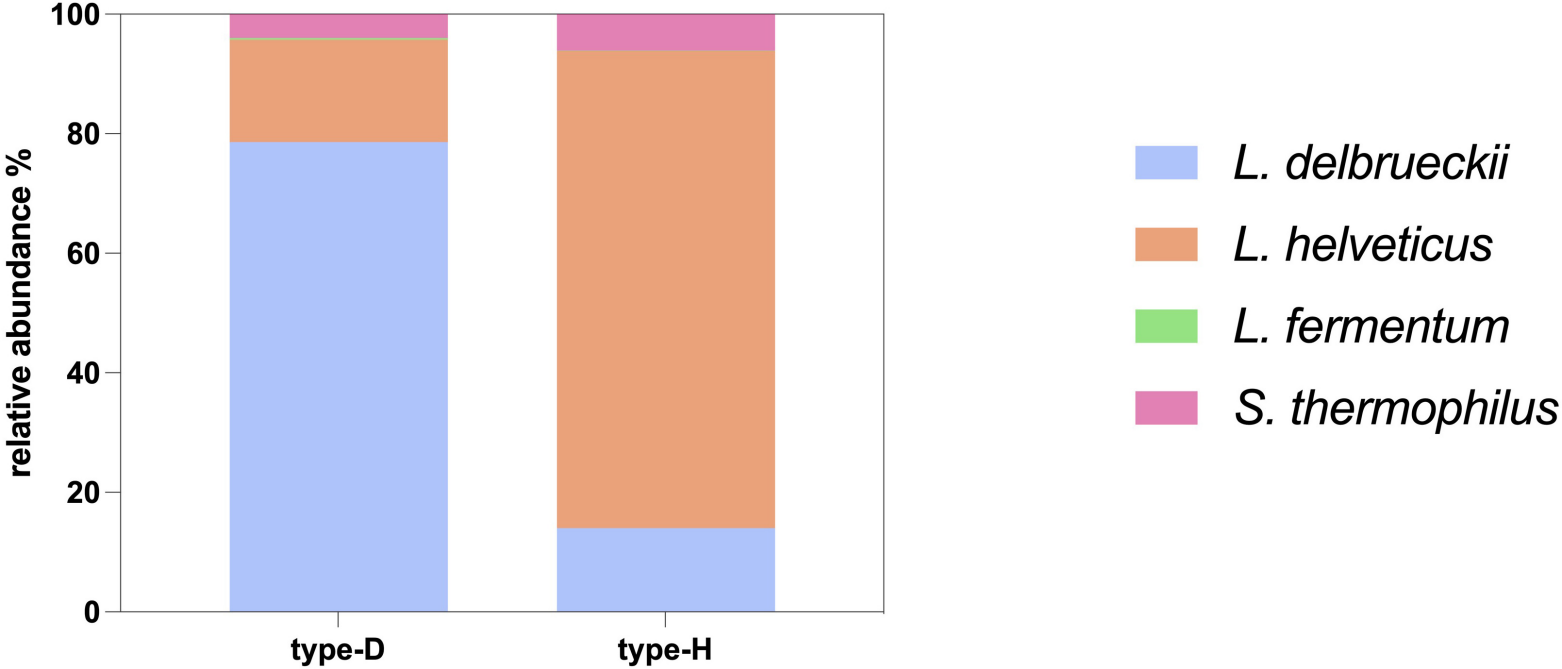
Taxonomic composition of type-D and type-H NWS assessed by metagenomics. Metagenomics results were expressed as average abundances of three biological replicates for each type of NWS.

Metataxonomic profiles were determined for type-D and type-H NWS samples. Both communities were dominated by *L. delbrueckii* and *L. helveticus*, cumulatively accounting for > 70% of the total microbial abundance. The type-D sample was enriched in *L. delbrueckii* (78.62%), followed by *L. helveticus* (17.14%), *S. thermophilus* (3.96%), and *L. fermentum* (0.28%), whereas the type-H NWS in *L*. *helveticus* (79.81%), followed by *L. delbrueckii* (14.03%), *S. thermophilus* (6.10%), and *L. fermentum* (0.06%) (Figure 2). The results were consistent with the previous classification of these NWS cultures in type-D and type-H communities (Sola et al., 2022).

### 3.2 Effect of different nitrogen sources on plate counts and cultivability of NWS lactobacilli

The reduced viability of the NWS lactobacilli may be related to nutritional limitations, such as auxotrophies for several amino acids and growth factors, which are commonly reported in LAB species. To evaluate this hypothesis, 11 different MRS-based media were tested for the ability to support NWS lactobacilli growth, assessed both by plate counts (Log_10_ CFU/mL) and by the capability to maintain isolates viable *ex situ* over time as axenic culture. Five nitrogen and growth factors sources were used as supplements: caseins hydrolysate (1, 2, and 4% w/v), tryptone (1, 2, and 4% w/v), lactoalbumin hydrolysate (1, 2, and 4%), YE (4% w/v), and SSW (7% w/v). For each supplementation, type-D NWS and one type-H NWS samples previously characterized by metagenomics were submitted to microbiological analyses.

**FIGURE 3:**
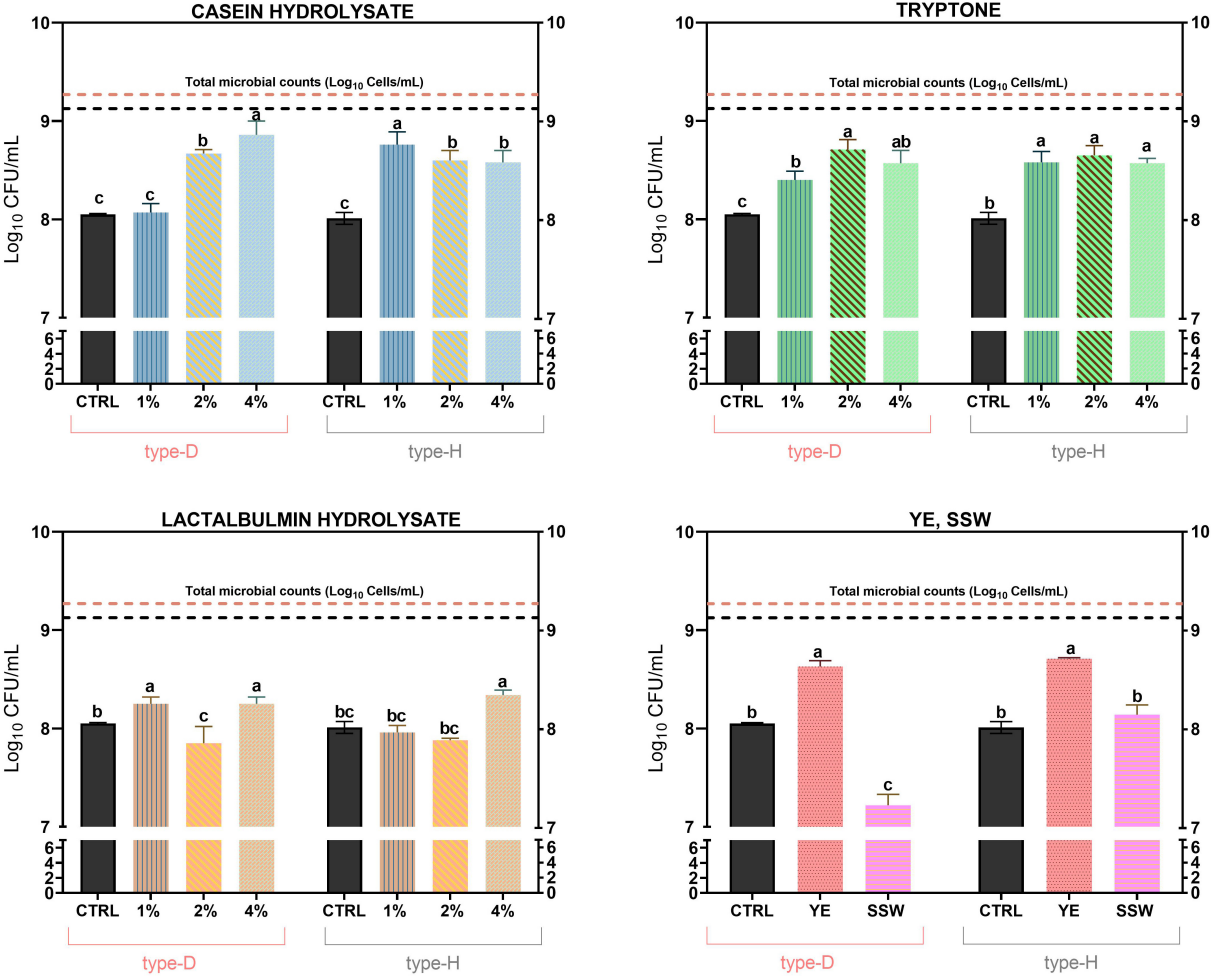
Effects of different nitrogen source supplementations on plate counts values (expressed as Log_10_ CFU/mL) of lactobacilli from type-D and type-H NWS. MRS medium (pH 6.5) was used as the control condition (CTRL). Values are the mean of at least three replicates and error bars represent standard deviation. Different letters indicate significantly different values (*p* < 0.05). Horizontal orange and black dotted lines represent total microbial counts (TMC) values (expressed as Log_10_ Cells/mL) of type-D and type-H NWS, respectively. YE, MRS supplemented with yeast extract; SSW, MRS supplemented with skimmed sweet whey.

Concerning plate counts, supplementations with CAS, YE, and TRYPTO were the most effective supplements in supporting the growth of NWS lactobacilli. Specifically, caseins supplementation increased plate counts compared to the control condition (MRS without supplementations; *p* < 0.05) (Figure 3A). In type-D NWS sample, this effect was concentration-dependent, with caseins 4% yielding the highest Log_10_ CFU/mL values, whereas in type-H sample, 1% caseins supplementation resulted in the highest plate counts (Figure 3A).

A similar trend was observed for tryptone supplementations. In the type-D sample, plate counts increased proportionally with tryptone concentration compared to the control (*p* < 0.05), whereas in the type-H sample, 1% tryptone was enough to significantly enhance plate counts relative to MRS medium (Figure 3B).

Lactalbumin hydrolysate also significantly improved plate counts compared to the control, but only at the highest concentrations tested in both type-D and type-H samples (Figure 3C).

By contrast, YE and SSW supplementations produced divergent effects: YE significantly increased plate counts compared to the control, whereas SSW supplementation failed to promote lactobacilli growth in both NWS samples (Figure 3D).

**FIGURE 4:**
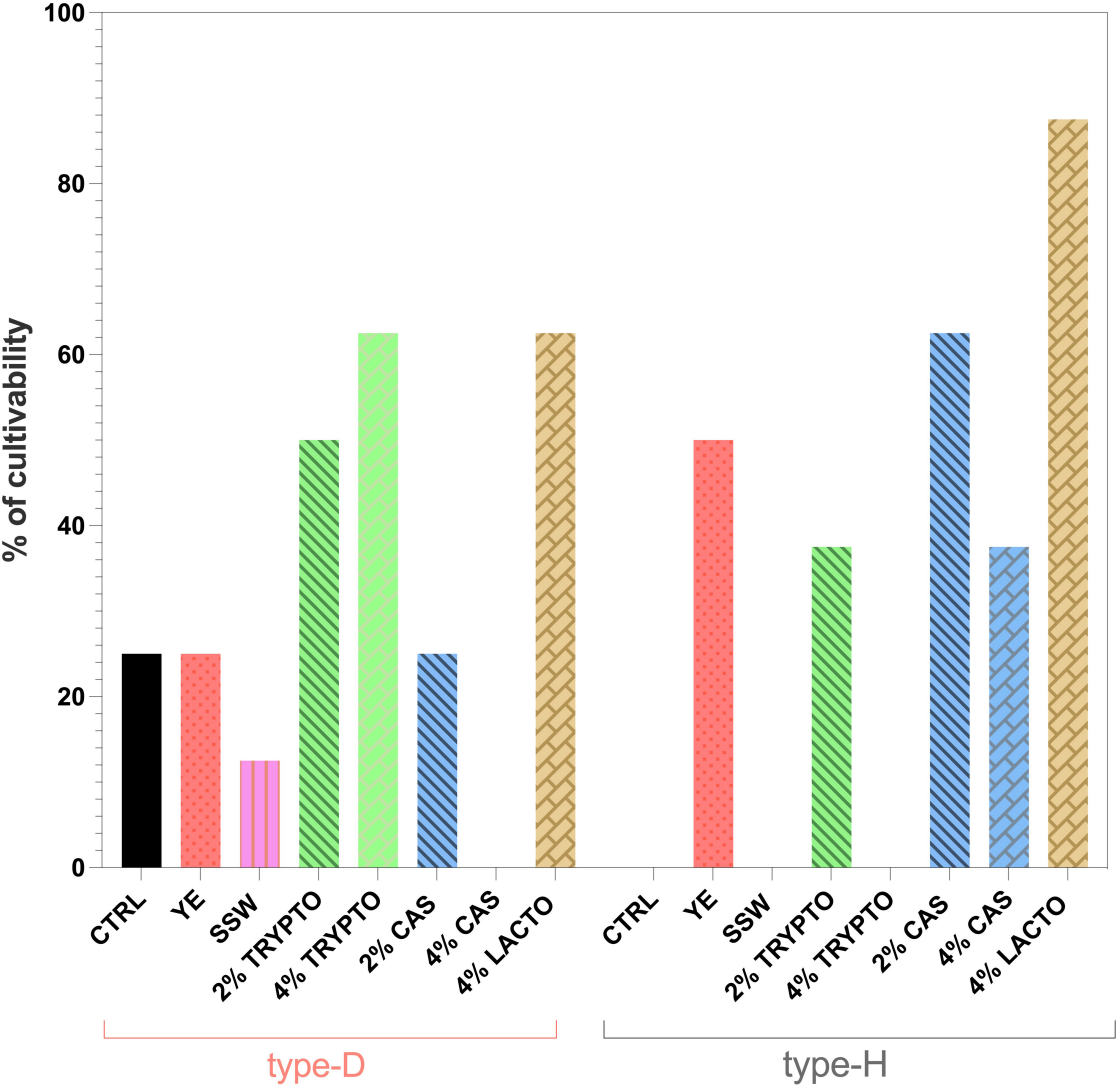
Effects of different nitrogen source supplementations on the cultivability of type-D and type-H NWS lactobacilli grown as axenic cultures. Cultivability is expressed as percentage of axenic cultures which remained viable and cultivable after three consecutive rounds of purification on the same medium. MRS medium (pH 6.5) was used as the control condition (CTRL). YE, MRS supplemented with yeast extract; SSW, MRS supplemented with skimmed sweet whey; 2% TRYPTO, MRS supplemented with 2% tryptone; 4% TRYPTO, MRS supplemented with 4% tryptone; 2% CAS, MRS supplemented with 2% casein hydrolysate; 4% CAS, MRS supplemented with 4% casein hydrolysate; 4% LACTO, MRS supplemented with 4% lactalbumin hydrolysate.

The 7 most effective supplements in supporting plate counts were subsequently selected to evaluate their capability to support cultivability of NWS lactobacilli, assessed as the percentage of viable axenic cultures after three successive purification steps. Remarkably, the supplements that have maximized plate counts were largely ineffective in sustaining the cultivability during axenic propagation (Figure 4). By contrast, MRS supplemented with 4% (v/v) lactalbumin hydrolysate resulted in the highest cultivability for isolates from both type-D and type-H NWS, although it failed to enhance plate counts. For isolates from type-D NWS, supplementation with 4% tryptone yielded cultivability levels comparable to those observed with 4% lactalbumin, whereas YE supplementation maintained cultivability in approximatively 20% of isolates, similar to the control condition. For the type-H NWS isolates, 4% lactalbumin resulted in the highest cultivability, followed by 2% caseins, and YE (Figure 4). Across most culture conditions, the highest mortality rate was observed during the first purification step for both the sets of isolates (data not shown).

### 3.3 Effect of different nitrogen sources on NWS species representativeness

To evaluate whether supplementation of MRS medium with different nitrogen sources affected species representativeness, isolates were identified at species level using DNA barcoding methods. As shown in Table 3, the majority of lactobacilli isolated from both type-D and type-H NWS samples were identified as *L. helveticus*, suggesting that all tested media largely failed to support the cultivation of *L. delbrueckii*, even when this species dominated the microbial community of type-D NWS, as revealed by metataxonomic analysis (Figure 2). Remarkably, only MRS-SSW and MRS-LACTO 4% enabled the isolation of a limited number of *L. delbrueckii* strains and maintained them in a viable and cultivable state. In contrast, MRS-YE supported the isolation of *L. helveticus* and *L. fermentum* only (Table 3). Nevertheless, none of the tested media reflected the actual species composition observed in type-D NWS (Figure 2). Taken together, the results indicate that supplementation strategies that effectively increased plate counts, such as MRS-YE, MRS-TRIPTO 2 and 4%, MRS-CAS 2 and 4%, were not suitable for cultivating *L. delbrueckii.* This outcome is likely attributable to the reduced survival of *L. delbrueckii* during subsequent purification steps, rather than to insufficient initial growth on these media.

**TABLE 3:** Effect of different nitrogen source supplementations on species representativeness of lactobacilli isolates from type-D and type-H NWS cultures.

Media	NWS type-D			NWS type-H		
	Lh (%)	Lf (%)	Ld (%)	Lh (%)	Lf (%)	Ld (%)
CTRL	71.43	28.57	0.00	100.00	0.00	0.00
YE	100.00	0.00	0.00	80.00	20.00	0.00
SSW	0.00	71.43	28.57	100.00	0.00	0.00
2% TRYPTO	42.86	57.14	0.00	100.00	0.00	0.00
4% TRYPTO	100.00	0.00	0.00	100.00	0.00	0.00
2% CAS	100.00	0.00	0.00	100.00	0.00	0.00
4% CAS	100.00	0.00	0.00	100.00	0.00	0.00
4% LACTO	75.00	12.50	12.50	100.00	0.00	0.00

Taxonomic attribution was assessed by culture-based DNA barcoding methods. Results were expressed as percentage of isolates assigned to a given species. MRS medium (6.5 pH) was used as control condition (CTRL). Lh, *L. helveticus*; Lf, *L. fermentum*; Ld, *L. delbrueckii* subsp. *lactis*; YE, MRS supplemented with yeast extract; SSW, MRS supplemented with skimmed sweet whey; TRYPTO 2%, MRS supplemented with 2% tryptone; TRYPTO 4%, MRS supplemented with 4% tryptone; CAS 2%, MRS supplemented with 2% caseins hydrolysate; CAS 4% MRS supplemented with 4% caseins hydrolysate; LACTO 4%, MRS supplemented with 4% lactalbumin hydrolysate, respectively.

### 3.4 Effect of cysteine, formic acid, and folate supplementations on plate counts and cultivability of NWS lactobacilli

As members of a complex microbial community, NWS lactobacilli may be poorly adapted to grow under axenic conditions, where microbial interactions and metabolite exchanges are absent. In Swiss cheese NWS cultures, formic acid and folate are produced by *S. thermophilus* and used by *L. delbrueckii* (Somerville et al., 2022). This metabolic interaction is also a well-known feature of the protocooperation between *S. thermophilus* and *L. delbrueckii* subsp. *bulgaricus* in yogurt cultures (Sieuwerts et al., 2008, 2010). Furthermore, cysteine supplementation has been reported to enhance the growth of several oxygen-sensitive lactobacilli (Dave and Shah, 1998; Soto et al., 2019; Serrador et al., 2025). Based on these considerations, the effects of cysteine, formic acid, and folate supplementations on plate counts and cultivability of NWS lactobacilli were investigated. Specifically, C medium, containing L-cysteine (0.05% w/v), CF medium, containing L-cysteine (0.05% w/v) and formic acid (5 mM), and CFFa medium, containing L-cysteine (0.05% w/v), formic acid (5 mM), and folic acid (226 μM), were evaluated for the ability both to support plate counts of NWS lactobacilli and to maintain isolates from both type-D and type-H NWS in a viable and cultivable state.

**FIGURE 5:**
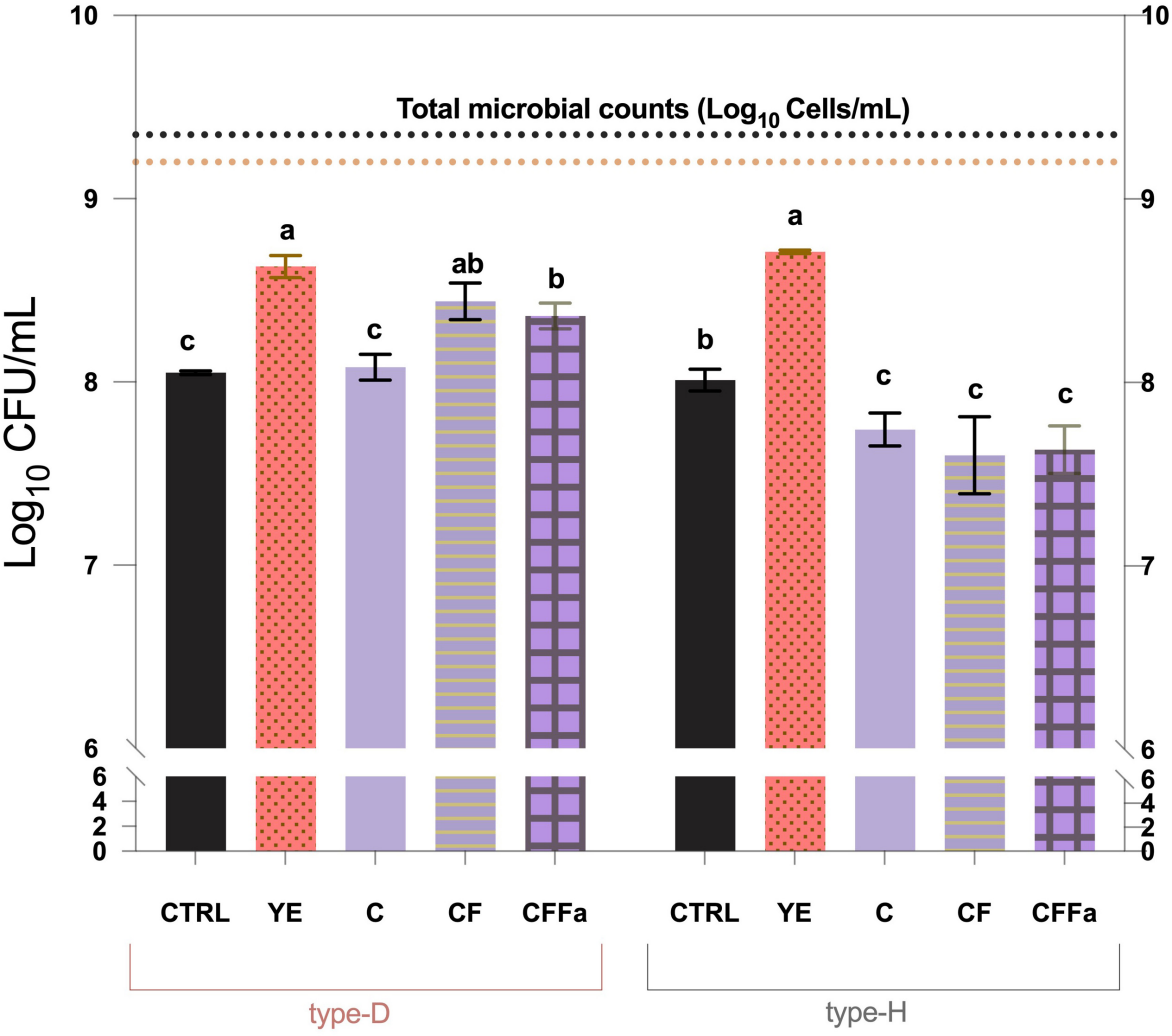
Effect of C, CF, and CFFa media on plate counts values (expressed as Log_10_ CFU/mL) of type-D and type-H NWS cultivable lactobacilli fractions. MRS medium (pH 6.5) was used as the control condition (CTRL). Values are the mean of at least three replicates and error bars represent standard deviation. Different letters indicate significantly different values (*p* < 0.05). Horizontal orange and black dotted lines represent total microbial counts (TMC) values (expressed as Log_10_ cells/mL) of type-D and type-H NWS, respectively. YE, MRS supplemented with yeast extract; C, MRS supplemented with cysteine; CF, MRS supplemented with cysteine and formic acid; CFFa, MRS supplemented with cysteine, formic acid, and folic acid.

MRS-YE medium was confirmed to be the most effective for plate count recovery in both NWS samples (*p* < 0.05) (Figure 5). By contrast, addition of cysteine, formic acid, and folic acid produced distinct effects in the two NWS samples. In type-D NWS, the highest plate counts were scored on CF medium, which exhibited values comparable with those observed on MRS and MRS-YE. Conversely, in type-H NWS, all tested conditions showed significantly lower Log_10_ CFU/mL values compared with the control MRS medium (*p* < 0.05) (Figure 5). These differences may reflect the distinct microbial composition of the two NWS cultures, with type-D NWS being characterized by a higher abundance of *L. delbrueckii*, and type-H NWS dominated by *L. helveticus* (Figure 2). The results suggest that supplementation with cysteine and formic acid may favor the growth of *L. delbrueckii*, which was present at high abundance in type-D NWS.

**FIGURE 6:**
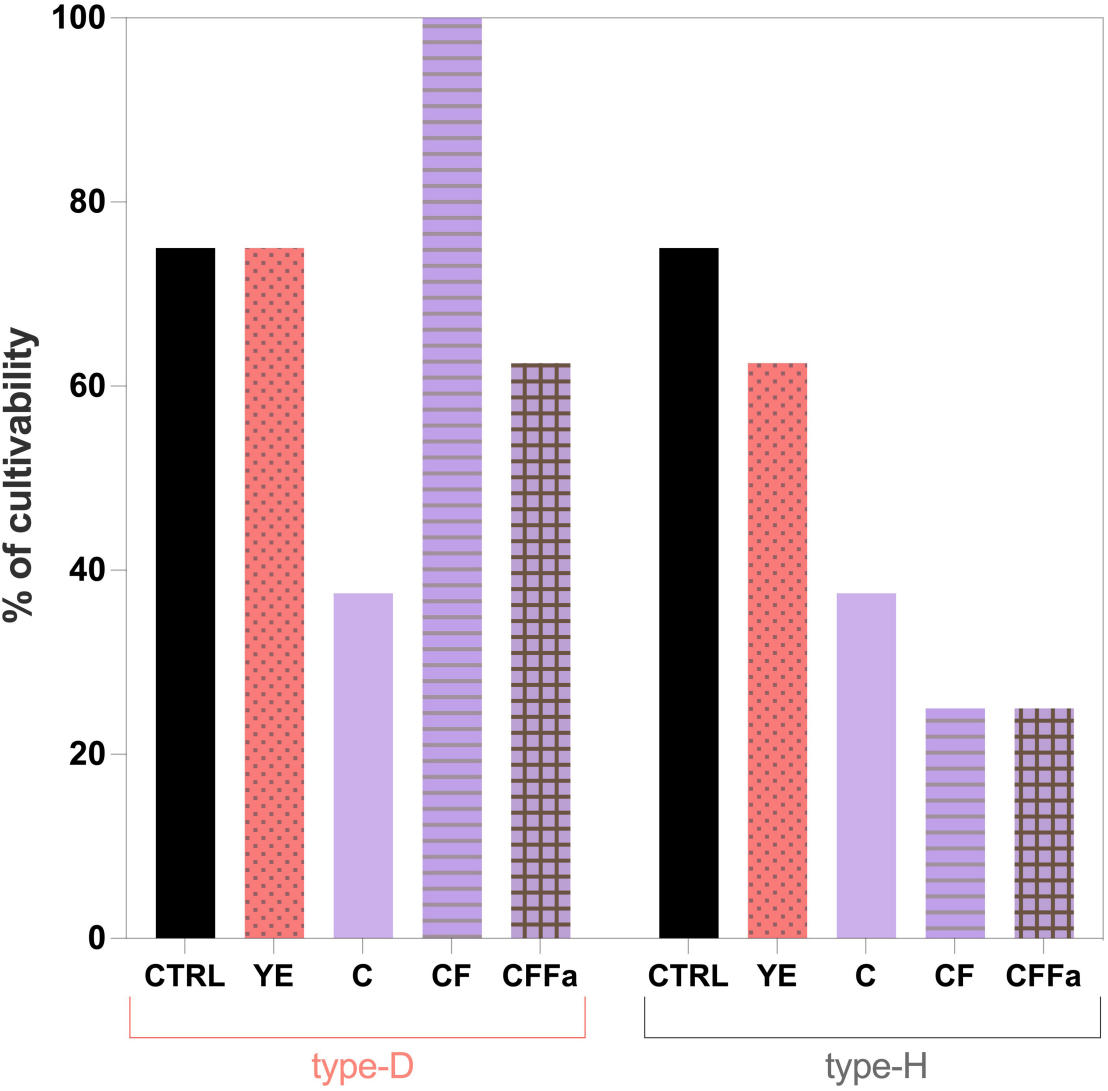
Effects of C, CF, and CFFa media on the cultivability of type-D and type-H NWS lactobacilli grown as axenic cultures. Cultivability is expressed as percentage of axenic cultures which remained viable and cultivable after three consecutive rounds of purification on the same medium. MRS medium (pH 6.5) was used as the control condition (CTRL). YE, MRS supplemented with yeast extract; C, MRS supplemented with cysteine; CF, MRS supplemented with cysteine and formic acid; CFFa, MRS supplemented with cysteine, formic acid, and folic acid.

Cultivability results were consistent with the trends observed for plate count recovery. As shown in Figure 6, supplementation with cysteine and formic acid resulted in 100% cultivability of isolates derived from type-D NWS. In contrast, for type-H NWS, cultivability values obtained with C, CF, and CFFa media were significantly lower than those observed with MRS and MRS-YE (*p* < 0.05). Overall, these findings support that supplementation with cysteine and formic acid represents the most effective condition for both enumeration and isolation of the lactobacilli fraction from type-D NWS enriched in *L. delbrueckii*.

### 3.5 Effect of cysteine, formic acid, and folic acid on NWS species representativeness

The ability of C, CF, and CFFa media to isolate the species present within type-D or type-H NWS communities was evaluated by performing species identification of axenic cultures obtained from each culture condition. For isolates derived from type-D NWS, CF medium was the only condition that supported the maintenance of *L. delbrueckii* isolates in a viable and cultivable state (Table 4). However, *L. delbrueckii* accounted for only 35% of the isolates recovered on CF medium, indicating that the dominance of *L. delbrueckii* has not been completely represented under this cultivation condition. Interestingly, all *L. delbrueckii* isolates belonged to the subspecies *lactis*, as determined by 16S-ARDRA with *Eco*RI (Giraffa et al., 1998) and confirmed by 16S rRNA gene sequencing. In contrast, for isolates obtained from type-H NWS, MRS-YE was the only medium that allowed the recovery of both *L. helveticus* and *L. fermentum*. None of the tested media allowed for the isolation of the minor *L. delbrueckii* fraction present in type-H NWS (Table 4).

**TABLE 4:** Effect of L-cysteine, formic acid, and folate on species representativeness of lactobacilli isolates from type-D and type-H NWS cultures.

Media	NWS type-D			NWS type-H		
	Lh (%)	Lf (%)	Ld (%)	Lh (%)	Lf (%)	Ld (%)
CTRL	80.00	20.00	0.00	100.00	0.00	0.00
YE	66.67	33.33	0.00	80.00	20.00	0.00
C	100.00	0.00	0.00	100.00	0.00	0.00
CF	62.50	0.00	37.50	100.00	0.00	0.00
CFFa	100.00	0.00	0.00	100.00	0.00	0.00

Taxonomic attribution was assessed by culture-based DNA barcoding methods. Results were expressed as percentage of isolates assigned to a given species. MRS medium (pH 6.5) was used as control condition (CTRL). Lh, *L. helveticus*; Lf, *L. fermentum*; Ld, *L. delbrueckii* subsp. *lactis*; YE, MRS supplemented with yeast extract; C, MRS supplemented with cysteine; CF, MRS supplemented with cysteine and formic acid; CFFa, MRS supplemented with cysteine, formic acid, and folic acid.

### 3.6 Effect of cysteine, formic acid, and folic acid on growth of *L. delbrueckii* subsp. lactis axenic cultures

To further confirm the positive effect of cysteine and formic acid supplementation on the growth of *L. delbrueckii* subsp. *lactis*, growth performance of two representative strains (C5I3 and T1104) was evaluated in SSW, C, CF, and CFFa media and expressed as a percentage relative to growth in MRS medium used as the control. While SSW supplementation decreased the growth percentage of both strains, C, CF, and CFFa exerted a positive effect, with C and CF representing the most effective conditions (*p* < 0.05) (Supplementary Figure 1).

### 3.7 Milk acidification trends in cross-feeding trials

To test whether the presence of other species could specifically promote the growth and activity of *L. delbrueckii*, cross-feeding experiments were carried out by fermenting milk with different combinations of mono-, co-, and tri-cultures of *L. helveticus*, *L. delbrueckii* subsp. *lactis*, and *S. thermophilus* strains isolated from type-D and type-H NWS communities. Specifically, *L. helveticus* (Lh) strains C001.15 and LBB04, *L. delbrueckii* subsp. *lactis* (Ld) strains T1104 and C5I3, and *S. thermophilus* strains (St) RBC06 and C001.27 were combined into three inoculation sets: set 1 (C001.15, RBC06, and T1104), set 2 (LBB04, C001.27, and C5I3), and set 3 (C001.15, RBC06, and C5I3) (Figure 1). Each strain was inoculated into milk as monocultures, in pairwise co-cultures, and tri-cultures. Acidification kinetics (Supplementary Figure 2) were analyzed to estimate the maximum acidification rate (μ_*max*_) and maximum acidification efficiency (ΔpH_*max*_).

**FIGURE 7:**
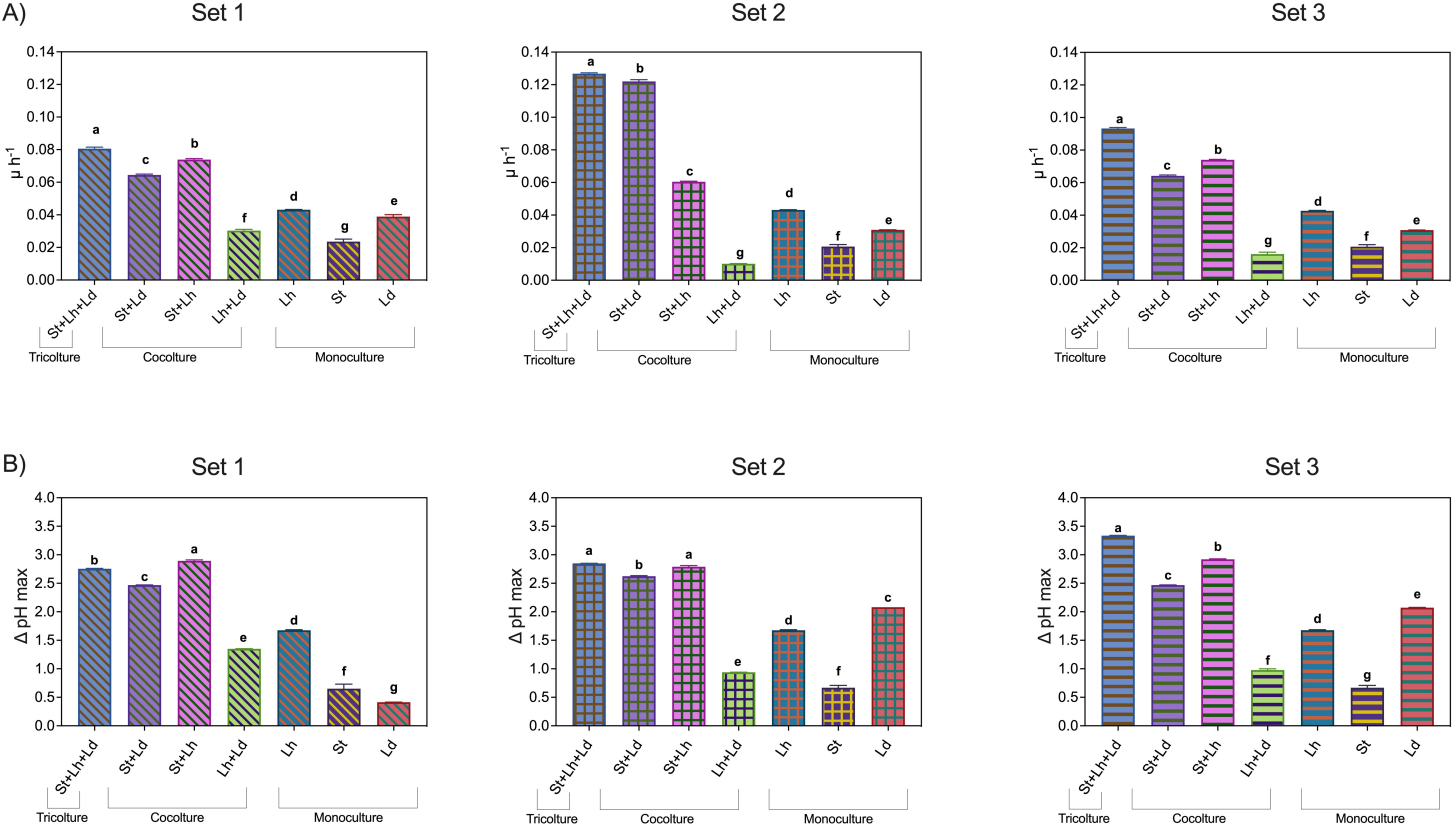
Maximum acidification rate (μ_max_, h^–1^) **(A)** and maximum fermentative efficiency (ΔpH_max_) **(B)** of *L. helveticus* (Lh), *L. delbrueckii* (Ld), and *S. thermophilus* (St) NWS strains in monoculture, co-culture, and tri-culture. Panels show set 1 (Lh C001.15, St RBC06, Ld T1104), set 2 (Lh LBB04, St C001.27, Ld C5I3), and set 3 (Lh C001.15, St RBC06, and Ld C5I3). Values represent means of three replicates ± SD. Different lowercase letters indicate statistically significant differences as evaluated through one-way ANOVA (*p* < 0.05).

In monoculture, Lh exhibited the highest acidification rates, followed by Ld and St, respectively (*p* < 0.05) (Figure 7A). Both Ld monocultures showed intermediate μ_*max*_ values relative to Lh and St monocultures (Figure 7A). Across all inoculation sets, tri-cultures consistently displayed the highest μ_*max*_ values (*p* < 0.05), followed by the co-cultures St + Lh and St + Ld.

Remarkably, in all strain combinations tested, co-inoculation of milk with Lh and Ld resulted in reduced acidification rates compared with the corresponding monocultures, regardless of the specific Ld and Lh strains used (Figure 7A). By contrast, in St + Ld co-cultures, μ_*max*_ values were significantly higher than those observed in the corresponding Ld and St monocultures (*p* < 0.05) (Figure 7A), indicating a mutual benefit from co-cultivation. A similar enhancement was observed in Lh + St co-cultures, where all strain combinations exhibited significantly higher acidification rates than their respective monocultures (*p* < 0.05) (Figure 7A). Interestingly, in inoculation set 2, co-cultivation of St strain C001.27 with Ld strain C5I3 resulted in μ_*max*_ values comparable to those observed under tri-culture conditions. In contrast, co-cultivation with Lh strain LBB04 yielded significantly lower μ_*max*_ values compared to the corresponding St + Ld interaction. However, these values remained comparable to those recorded for the co-culture condition in other inoculation sets (*p* < 0.05) (Figure 7A).

Statistical analysis of the maximum acidification efficiency (ΔpH_*max*_) largely confirmed the trends described for μ_*max*_, with the exception of Ld strain T1104 in inoculation set 1 (Figure 7B). Strain T1104 showed lower ΔpH_*max*_ than the conspecific strain C5I3 and exhibited a such limited acidification ability in monoculture that co-cultivation with Lh strain C001.15 resulted in lower final pH values and, consequently, a higher ΔpH_*max*_ compared with the T1104 monoculture (*p* < 0.05). Nonetheless, even under these conditions, Lh + Ld co-cultures remained less effective in acidification than Lh monoculture (*p* < 0.05).

Overall, the findings support the presence of negative interactions between *L. helveticus* and *L. delbrueckii* subsp. *lactis* and positive interactions between *L. delbrueckii* subsp. *lactis* and *S. thermophilus*, as well as between *S. thermophilus* and *L. helveticus*.

### 3.8 D- and L-lactate production in cross-feeding trials

Since *S. thermophilus* mainly produces L-lactate (Ghailan and Niamah, 2025), *L. delbrueckii* subsp. *lactis* produces D-lactate (Bernard et al., 1991), and *L. helveticus* produces a racemic mixture of L- and D-lactate (Kylä-Nikkilä et al., 2000), the D/L-lactate ratio at the end of milk fermentation can provide insights into species interactions during co-cultivation. Accordingly, milk samples collected at the end of each fermentation were analyzed to determine D- and L-lactate concentrations.

**FIGURE 8:**
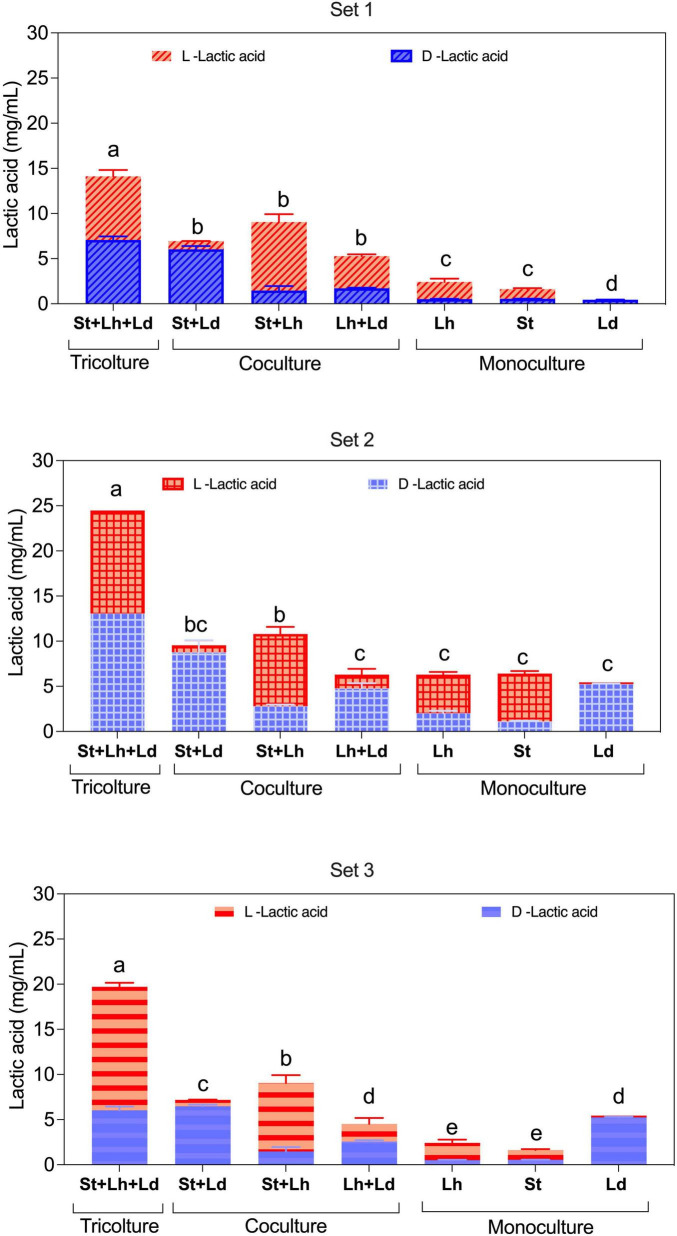
L-lactate (red) and D-lactate (blue) concentrations (expressed as mg/mL) detected at the end of milk fermentation with *L. helveticus* (Lh), *L. delbrueckii* (Ld), and *S. thermophilus* (St) NWS strains in monoculture, co-culture, and tri-culture. Panels show set 1 (Lh C001.15, St RBC06, Ld T1104), set 2 (Lh LBB04, St C001.27, Ld C5I3), and set 3 (Lh C001.15, St RBC06, and Ld C5I3). Values represent means of three replicates ± SD. Different lowercase letters indicate statistically significant differences in total lactate concentrations, as evaluated through one-way ANOVA (*p* < 0.05).

In monoculture, Lh strains produced a racemic mixture of lactic acid, whereas Ld strains produced exclusively D-lactate, as expected (Figure 8). However, the limited growth of Ld strain T1104 resulted in a very low level of D-lactate. St strains RBC06 and C001.27 predominately produced L-lactate, which accounted for 67.2 and 83% of total lactate, respectively. Regardless of the lactate enantiomer produced, monocultures produced a very low amount of total lactate, consistent with the slow acidification phenotype previously reported for NWS isolates (Morelli et al., 1986; Fortina et al., 1998; Hebert et al., 2001).

Across all three strain combinations, total lactic acid production was highest in tri-cultures, followed by St + Lh and St + Ld co-cultures (*p* < 0.05) (Figure 8), consistent with the acidification kinetics. Although absolute lactate concentrations varied among inoculation sets, D-lactate and L-lactate were produced in comparable amounts in all tri-cultures, indicating that Ld strains remained viable and metabolically active. In two of the three sets (sets 1 and 2), D-lactate levels in tri-cultures were significantly higher than those detected in the corresponding Ld monocultures (*p* < 0.05), supporting the presence of positive interactions that enhanced Ld activity in mixed cultures.

In milk fermented with St and Lh, total lactate production increased relative to monocultures (Figure 8). Under St + Lh conditions, L-lactate predominates, accounting for 86.73% of total lactate in the co-culture of strains RBC06 and C001.5 and 74.08% in the co-culture of strains C001.27 and LBB04.

Similarly, in St + Ld co-cultures, total lactate concentrations also exceeded those of the respective monocultures, with production dominated by D-lactate, suggesting that acidification was largely attributable to Ld activity (Figure 8).

In inoculation sets 2 and 3, Lh + Ld co-cultures yielded significantly lower total lactic acid than tri-cultures and the St + Lh and St + Ld co-cultures (Figure 8). In both cases, D-lactate accounted for the majority of lactate produced (75.95% in set 2 and 56.63% in set 3, respectively), indicating that Ld was metabolically active. Consistent with ΔpH_*max*_ values, an exception was observed in inoculation set 1, where the Lh + Ld co-culture, represented by C001.15 and T1104, produced total lactate amount comparable to those of St + Lh and St + Ld co-cultures (*p* > 0.05) (Figure 8), but significantly lower than the corresponding tri-culture (*p* < 0.05) (Figure 8). In this Lh + Ld combination, L-lactate represented 67.42% of total lactate, suggesting that Lh was more metabolically active than Ld. This result is likely attributable to the very slow acidification phenotype of Ld strain T1104.

## 4 Discussion

Culture-independent approaches have substantially advanced our understanding of the ecology and evolution of microbial communities. Nevertheless, the isolation of individual strains remains indispensable for rigorous functional studies and for the development of reliable biotechnological applications (Zheng et al., 2024). In PR NWS, most culture-dependent studies have focused primarily on *L. helveticus* (Gatti et al., 2003), whereas *L. delbrueckii* subsp. *lactis* has received comparatively little attention. This gap is notable, as *L. delbrueckii* dominates specific PR NWS communities classified as type-D (Sola et al., 2022). Consequently, the ability to cultivate both dominant species is essential to experimentally validate metagenome-based inferences on microbial composition and interactions.

Although *L. delbrueckii* subsp. *lactis* has been isolated from Grana Padano NWS (Rossetti et al., 2008), several studies have reported difficulties in recovering this species from NWS and cheeses matrices using standard culture-based approaches (Randazzo et al., 2002; Monfredini et al., 2012; Morandi et al., 2019). According to these findings, our previous attempts to isolate *L. delbrueckii* using conventional MRS-based media were largely unsuccessful (Sola et al., 2022; Martini et al., 2024), thereby limiting the establishment of a representative NWS strain collection for controlled interactions studies. The present study therefore aimed to evaluate the impact of different nitrogen sources, as well as of cysteine, formic acid, and folic acid supplementations, on the recovery and maintenance of *L. delbrueckii* subsp. *lactis*, with the broader goal of exploring the possible contribution of microbial interactions to its impaired cultivability.

Culture media were evaluated according to three criteria: (i) recovery of plate counts, (ii) maintenance of cultivability during strain purification and biobanking, and (iii) ability to reflect species abundances within mixed communities. Our results demonstrated that none of nitrogen supplements tested fulfilled all three criteria simultaneously. Although several nitrogen supplements increased overall plate counts, they did not sustain cultivability during sub-culturing and failed to preserve the relative abundance of *L. delbrueckii*, suggesting that simple autotrophies for amino acids and other growth factors are unlikely to fully explain the poor *L. delbrueckii* cultivability. In particular, MRS-YE, commonly considered the elective medium for enumerating NWS lactobacilli (Reverberi et al., 2006), selectively supported *L. helveticus* and, to a lesser extent, *L. fermentum*, even when *L. delbrueckii* dominated the NWS community, thereby altering the species ratio. Similar trends were observed for tryptone- and caseins-based media. These results disagreed with previous studies reporting the enrichment of *L. delbrueckii* and *S. thermophilus* in YE-supplemented whey systems (Santarelli et al., 2008). The high cell viability in both type-H and type-D NWS samples suggested that *L. delbrueckii* is likely physiologically active within the NWS ecosystem, while the culture conditions tested here were insufficient to support its growth under axenic conditions.

Successful cultivation of microorganisms from complex ecosystems often depends on closely mimicking natural environmental conditions (Stewart, 2012). In line with this concept, whey-based media have been shown to improve the recovery of NWS bacteria (Gatti et al., 2003; Fornasari et al., 2006). In this study, MRS media supplemented with SSW or lactalbumin hydrolysate enabled limited recovery of *L. delbrueckii* isolates; however, neither formulation increase plate counts, limiting their practical applicability for large-scale biobanking.

The loss of cultivability observed for isolates from type-D NWS during recursive sub-culturing on MRS-YE, MRS-CAS, MRS-TRYPTO, and MRS-SSW suggests that *L. delbrueckii* may initially grow under these conditions but subsequently lose the ability to form colonies and died. One possible explanation is the induction of a viable but non-culturable (VBNC) state, which is characterized by a progressive loss of colony-forming ability on solid media under sublethal stress (Liu et al., 2023). Oxidative stress, including reactive oxygen species (ROS) generated during the autoclaving of nutrient-rich media (Tanaka et al., 2014), may reduce cultivability, particularly given that *L. delbrueckii* has been reported to produce hydrogen peroxide under microaerophilic conditions (Marty-Teysset et al., 2000). However, the VBNC state was not directly assessed in this study and therefore remains a working hypothesis rather than a demonstrated mechanism.

Cysteine supplementation, which provides redox buffering capacity, did not by itself enhance the recovery of *L. delbrueckii* from NWS cultures, whereas its combination with formic acid (CF) enabled the isolation of viable *L. delbrueckii* subsp. *lactis* strains from type-D NWS. In yogurt fermentation, formic acid, produced by *S. thermophilus* via pyruvatelyase, acts as a key growth factor for *L. delbrueckii* subsp. *bulgaricus* as it serves as a substrate for formyltetrahydrofolate synthetase in purine biosynthesis (Sieuwerts et al., 2008, 2010; Smid and Lacroix, 2013; Yamauchi et al., 2023). Repeated back-slopping of whey during NWS propagation may have selected for *L. delbrueckii* strains adapted to metabolite exchange within microbial consortia rather than to independent growth under axenic conditions. In line with this hypothesis, metagenome-assembled genomes (MAGs) analyses of Swiss hard cheese undefined starter cultures have reported gene loss in *L. delbrueckii*, including the absence of a complete purine biosynthesis pathway, whereas *S. thermophilus* MAGs retain folate and formic acid biosynthetic pathways which may benefit *L. delbrueckii* (Somerville et al., 2024). In addition, extensive pseudogenization driven by mobile genetic elements has been described in *L. delbrueckii* subsp. *lactis*, suggesting a propensity for functional gene loss in this species (Baek et al., 2023). Our findings are partially consistent with these observations, as the cysteine-formic acid (CF) combination enabled the recovery of viable *L. delbrueckii* sub. *lactis* from the type-D NWS. However, metagenomics studies will be required to determine whether genome decay occurs in *L. delbrueckii* from PR NWS cultures and contributes to the impaired cultivability of this species.

Some bacteria can only grow in a pure medium when in co-culture with another community member, also called a helper strain. Co-culturing can be achieved either by direct culturing of the helper strain together with the bacterium of interest or by using spent supernatants as a proxy for the helper strain (Stewart, 2012). In this study, co-cultivation experiments supported positive interactions between *L. delbrueckii* and *S. thermophilus*, as well as between *L. helveticus* and *S. thermophilus*, although the magnitude of these effects was strain-dependent.

In Ld + St co-cultures, positive interactions partially resemble the well-characterized protocooperation observed in yogurt fermentation (Sieuwerts et al., 2008). In this system, *S. thermophilus* ferments rapidly but typically slows its activity around pH 5, whereas *L. delbrueckii* becomes metabolically active at lower pH values and sustains fermentation over a longer period. *L. delbrueckii*, which is generally described as strongly proteolytic, can release peptides and amino acids that support the growth of the otherwise weakly proteolytic *S. thermophilus* (Giraffa et al., 2004; Savijoki et al., 2006; Sieuwerts et al., 2008; El Kafsi et al., 2014). In return, *S. thermophilus* produces metabolites such as folic acid, formic acid, and fatty acids that stimulate *L. delbrueckii* growth (Yang et al., 2025). In addition, urease and NADH oxidase activities of *S. thermophilus* may indirectly benefit *L. delbrueckii* by releasing ammonia and CO_2_ (raising pH) and by converting harmful H_2_O_2_ into water (Zotta et al., 2008; Arioli et al., 2017). However, unlike the mutualistic interactions described for yogurt cultures, the predominance of D-lactate over L-lactate observed in our Ld + St co-cultures suggest that, under the tested conditions, the interaction may preferentially enhance *L. delbrueckii* activity. From a cheesemaking perspective, altered lactate stereoisomer balance may influence downstream microbial metabolism during ripening and potentially affect texture and quality development in cooked hard cheeses. This aspect warrants further investigations.

When grown in monoculture, *L. helveticus* strains exhibited higher acidification capacity than *S. thermophilus* and *L. delbrueckii*, consistently with previous reports (Giraffa et al., 2004; Santarelli et al., 2013; El Kafsi et al., 2014). Like Ld + St co-cultures, co-cultures of *L. helveticus* and *S. thermophilus* also exhibited enhanced acidification compared with monocultures, although the molecular bases of this interaction are not yet fully understood. *L. helveticus* is unable to synthesize folic acid (Rossi et al., 2011) and is typically urease-negative (Zotta et al., 2008) and may therefore benefit from folic acid provision and urease activity of *S. thermophilus*. Conversely, *L. helveticus* exhibits strong proteolytic activity (Griffiths and Tellez, 2013), which may provide *S. thermophilus* with short peptides and amino acids. Together, these complementary traits may partially explain the positive interactions observed in Lh + St co-cultures; however, further studies are required to verify the underlying mechanisms.

In contrast to the positive interactions observed with *S. thermophilus*, our findings strongly suggest the absence of positive interactions between *L. helveticus* and *L. delbrueckii* subsp. *lactis*, under the conditions tested. The presence of *L. helveticus* did not enhance the acidification activity of *L. delbrueckii* and, conversely, *L. delbrueckii* did not act as a helper strain for *L. helveticus*. This lack of growth stimulation may reflect competition for available nutrients, as previously suggested in cheesemaking experiments (Charlet et al., 2009). Moreover, several studies report that antimicrobial molecules produced by these species can mediate negative interactions (Toba et al., 1991; Joerger and Klaenhammer, 1990; Van De Guchte et al., 2001). Giraffa et al. (1996) showed that spent supernatants of Lh inhibited the growth of Ld isolates cultured on reconstituted sweet whey in a strain-dependent manner, suggesting that inhibitory factors present in the whey supernatants may negatively affect co-occurring strains. Alternatively, strain-specific nutritional requirements which are not fulfilled in milk or whey may lead to differential growth behavior when Ld and Lh strains were co-cultivated on the same substrate.

Regardless of the underlying mechanism, the absence of positive interactions between *L. helveticus* and *L. delbrueckii*, together with strain-specific nutritional behaviors, may contribute to the emergence and maintenance of distinct NWS communities, as recently proposed by Sola et al. (2022). The extensive NWS strain biobank established in this study represents a valuable resource for future investigations using simplified microbial consortia mimicking type-D and type-H NWS, aimed at elucidating how different community configurations are established and how they influence fermentation dynamics and the sensory properties of PR cheese.

## 5 Conclusion

In conclusion, this study investigated the effects of 14 different supplementations on cultivability of *L. delbrueckii* subsp. *lactis* strains from PR NWS. Among the tested conditions, MRS supplemented with cysteine and formic acid (CF) was the only medium that supported the cultivability of a fraction of *L. delbrueckii* subsp. *lactis* isolates from type-D NWS, which was enriched in *L. delbrueckii* based on metataxonomic profiling. This finding was consistent with cross-feeding experiments demonstrating that co-cultivation of *L. delbrueckii* with the formate-producing species *S. thermophilus* significantly enhanced the milk acidification compared with monocultures. Together, these findings indicate that the impaired cultivability of *L. delbrueckii* subsp. *lactis* can be partially alleviated under conditions that mimic metabolic interactions with *S. thermophilus*, whereas no comparable effect was observed in co-culture with *L. helveticus* or under most axenic culture conditions tested. Overall, this study highlights the importance of microbial interactions in shaping the cultivability and functional behavior of *L. delbrueckii* subsp. *lactis* within NWS ecosystems and provides a basis for improved isolation strategies and future investigations into the role of this species in PR cheesemaking.

## Data Availability

Metataxonomic profiles datasets generated for this study have been deposited in the NCBI GenBank database under the BioProject accession number PRJNA1367500.
