# Supplementary material for: Role of microbial interactions in the impaired cultivability of thermophilic lactic acid bacteria in natural whey starter for Parmigiano Reggiano PDO cheese production
Source: Front Microbiol. 2026 Mar 4;17:1755652. doi: 10.3389/fmicb.2026.1755652 (PMC12996222; doi:10.3389/fmicb.2026.1755652)
Supplement: Supplementary file 1 [file Data_Sheet_1.docx]

**Role of Microbial Interactions in the Impaired Cultivability of Thermophilic Lactic Acid Bacteria in Natural Whey Starter for Parmigiano Reggiano PDO Cheese Production**

Supplementary Data

## Supplementary Figures


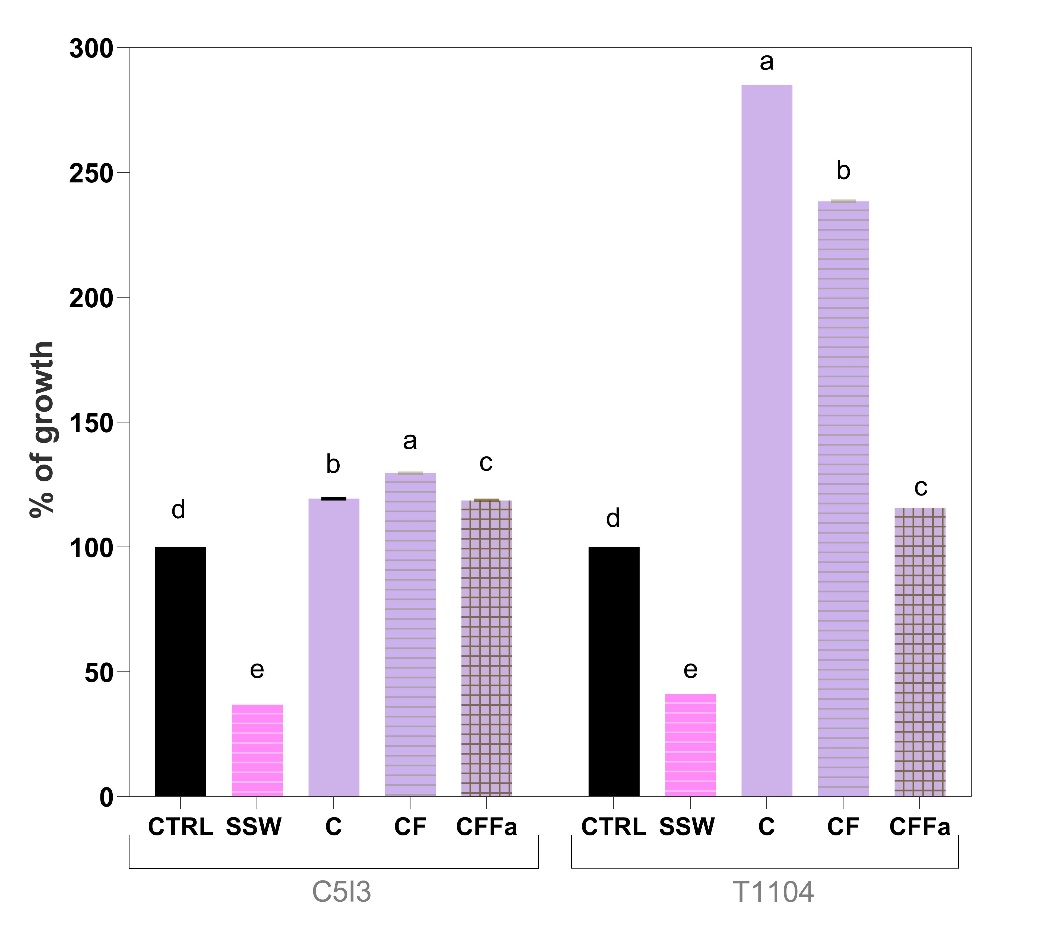


**Supplementary Figure S1**. Growth percentages of *Lactobacillus delbrueckii* subsp. *lactis* strains C5I3 and T1104 in MRS medium supplemented with sterile skimmed whey (SSW), L-cysteine (C), L- cysteine and formic acid (CF), and L-cysteine, formic acid, and folate (CFFa). Optical density at 600 nm (OD₆₀₀) was measured after 72 h of incubation at 42 °C under anaerobic conditions. Data are expressed as the percentage of growth relative to that obtained in MRS medium, which served as the control condition (CTRL). Experiments were performed in triplicates. Bars, when visible, represent standard deviations (SD). Statistically significant differences (*p*<0.05) were determined with one-way ANOVA relative to the control condition and are indicated with different lowercase letters.

**
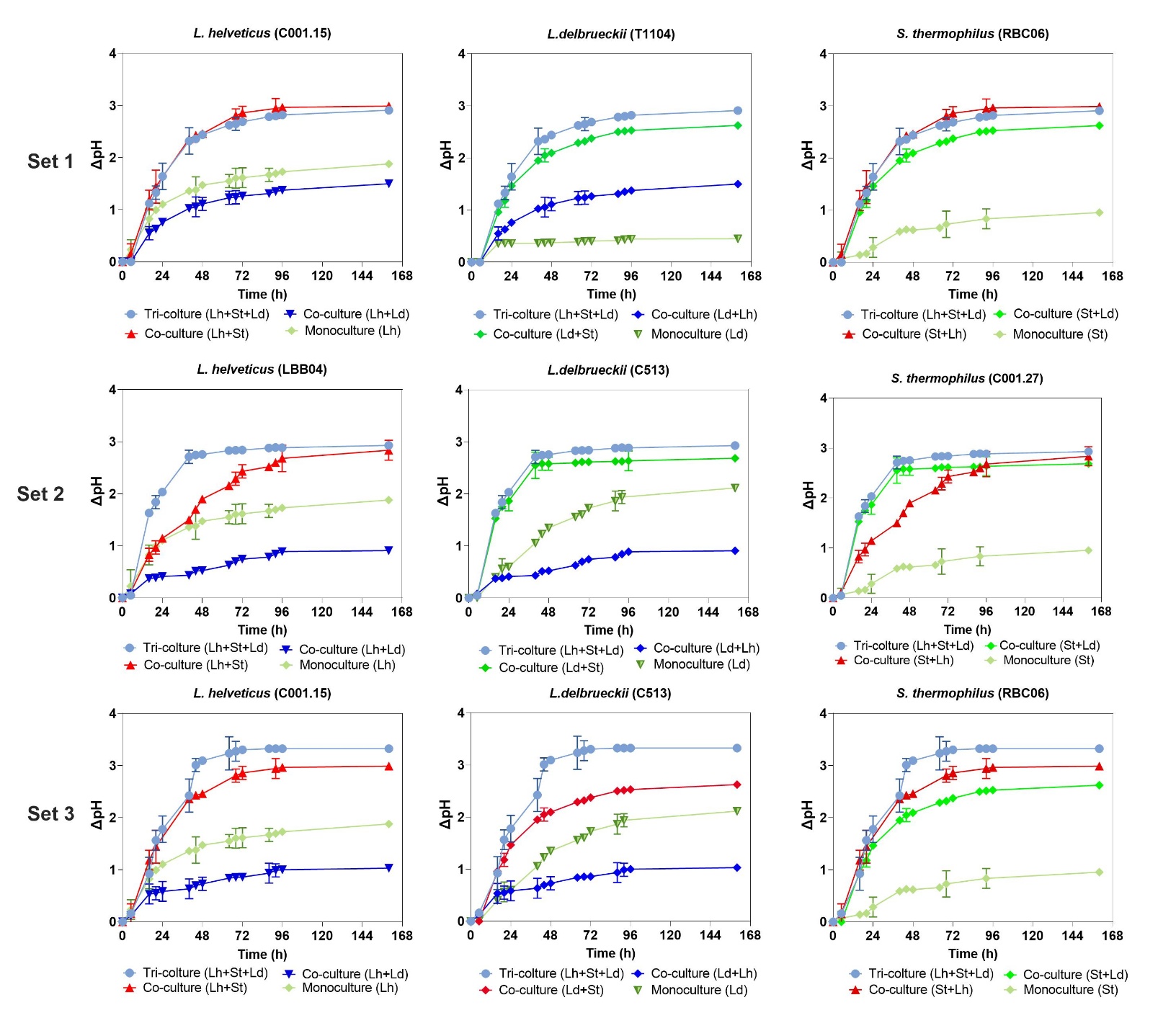
**

**Supplementary Figure S2**. Growth kinetics of three NWS strains combinations of *L. helveticus* (Lh), *L. delbrueckii* (Ld), and *S. thermophilus* (St) (namely, set 1, set 2, and set 3) inoculated as monocultures, co-cultures and tri-cultures. ΔpH (pH_zero time_ – pH_at each time_) values were measured over time, and data represent the mean values from three independent biological replicates. Error bars indicate standard deviation (SD). Set 1 included strains C001.15 (Lh), RBC06 (St), and T1104 (Ld); set 2 included LBB04 (Lh), C001.27 (St), and C5I3 (Ld); set 3 included C001.15 (Lh), RBC06 (St), and C5I3 (Ld). As a result, 3 tri-cultures, 6 monocultures, 6 co-cultures conditions were tested. Growth curves were fitted using the gcFitSpline function available in *grofit* R package for calculating maximum growth rate and maximum acidification (Kahm et al., 2010).

## Supplementary Tables

| **Strain code** | **Species** | **Isolation details (year of isolation; medium; temperature; w/wo O_2_; time)** | **Accession N.** | **Reference** |
| --- | --- | --- | --- | --- |
| RBC06 | *S. thermophilus* | 2020; M17-SSW; 42°C; w O_2_; 48-72 h | OM891849.1 | Solieri et al., (2022) |
| C001.27 | *S. thermophilus* | 2024; M17-SSW; 42°C; w O_2_; 48-72 h | PX617460 | this work |
| LBB04 | *L. helveticus* | 2020; MRS (pH 5.4); 42°C; wo O_2_; 48-72 h | ON936798.1 | Sola et al., (2022) |
| C001.15 | *L. helveticus* | 2024; YE; 42°C; wo O_2_; 48 h | PX617461 | this work |
| T1104 | *L. delbrueckii* subsp. *lactis* | 2021; M17-SSW, 42°C; wo O_2_; 72 h | ON755078.1 | Sola et al., (2022) |
| C5I3 | *L. delbrueckii* subsp. *lactis* | 2021; M17-SSW; 30°C; wo O_2_; 72 h | PX617462 | this work |

**Supplementary Table S1.** NWS strains used in this study and their isolation conditions. Accession N. refers to the accession number of 16S rRNA gene sequences deposited in Genbank. Abbreviations: YE, MRS medium supplemented with yeast extract; M17-SSW, M17 medium supplemented with skimmed sweet whey.
